# Harmonized geospatial data to support infrastructure siting feasibility planning for energy system transitions

**DOI:** 10.1038/s41597-023-02694-y

**Published:** 2023-11-09

**Authors:** Chris R. Vernon, Kendall Mongird, Kristian D. Nelson, Jennie S. Rice

**Affiliations:** Pacific Northwest National Laboratory, Richland, WA USA

**Keywords:** Energy and society, Energy access

## Abstract

Climate change, energy system transitions, and socioeconomic change are compounding influences affecting the growth of electricity demand. While energy efficiency initiatives and distributed resources can address a significant amount of this demand, the United States will likely still need new utility-scale generation resources. The energy sector uses capacity expansion planning models to determine the aggregate need for new generation, but these models are typically at the state or regional scale and are not equipped to address the wide range of location- and technology-specific issues that are increasingly a factor in power plant siting. To help address these challenges, we have developed the Geospatial Raster Input Data for Capacity Expansion Regional Feasibility (GRIDCERF) data package, a high-resolution product to evaluate siting suitability for renewable and non-renewable power plants in the conterminous United States. GRIDCERF offers 264 suitability layers for use with 56 power plant technologies in a harmonized format that can be easily ingested by geospatially-enabled modeling software allowing for customization to robustly address science objectives when evaluating varying future conditions.

## Background & Summary

Electricity demand in the United States (U.S.) is projected to increase due to climatic, socioeconomic, and decarbonization policy drivers^1–4^. Meeting these new demands will require siting new electricity system infrastructure (i.e., generation, transmission, and distribution) while addressing natural resource-, policy-, and technology-based challenges. For example, water stress (due to high water temperatures and/or reduced availability) is projected to negatively impact 27% of thermoelectric power production across the U.S. by 2030^5^. Other studies have highlighted the importance of considering local land conservation planning to address potential negative effects of energy system transitions on natural resources or biodiversity^6,7^. Interactions between technology change (e.g., the advent of small modular nuclear reactors), climate change (e.g., water availability), and socioeconomic change (e.g., changes in population density and demographics) may also affect power plant siting choices^8^. All these factors point to the need for a comprehensive, exploratory modeling approach to evaluate the impacts of siting feasibility on alternative electricity system capacity expansion plans^9^. To help address this challenge, we introduce the open-source GRIDCERF (Geospatial Raster Input Data for Capacity Expansion Regional Feasibility) data package.

**Fig. 1:**
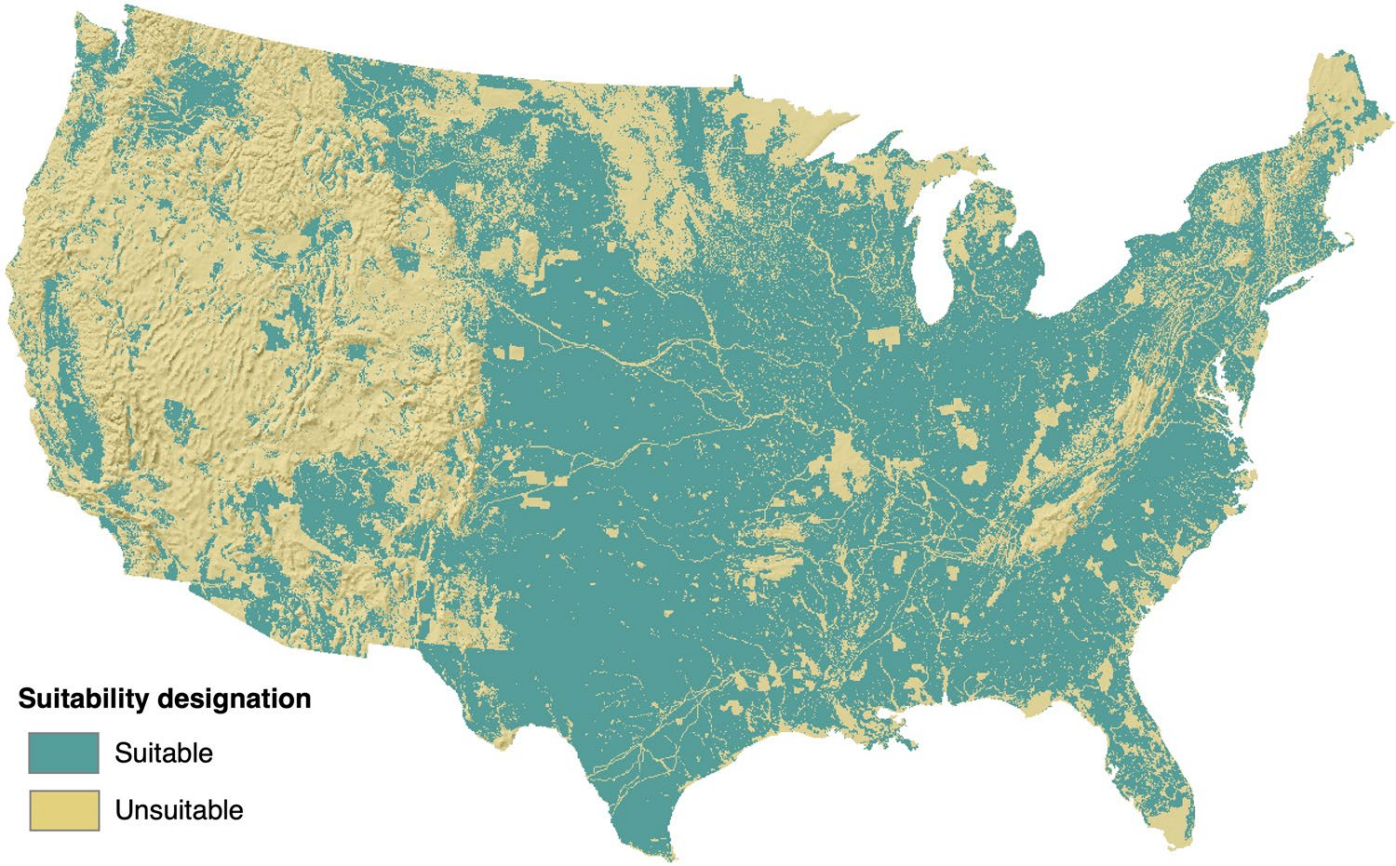
Combined suitability for layers common to all thermoelectric power plants.

**Fig. 2:**
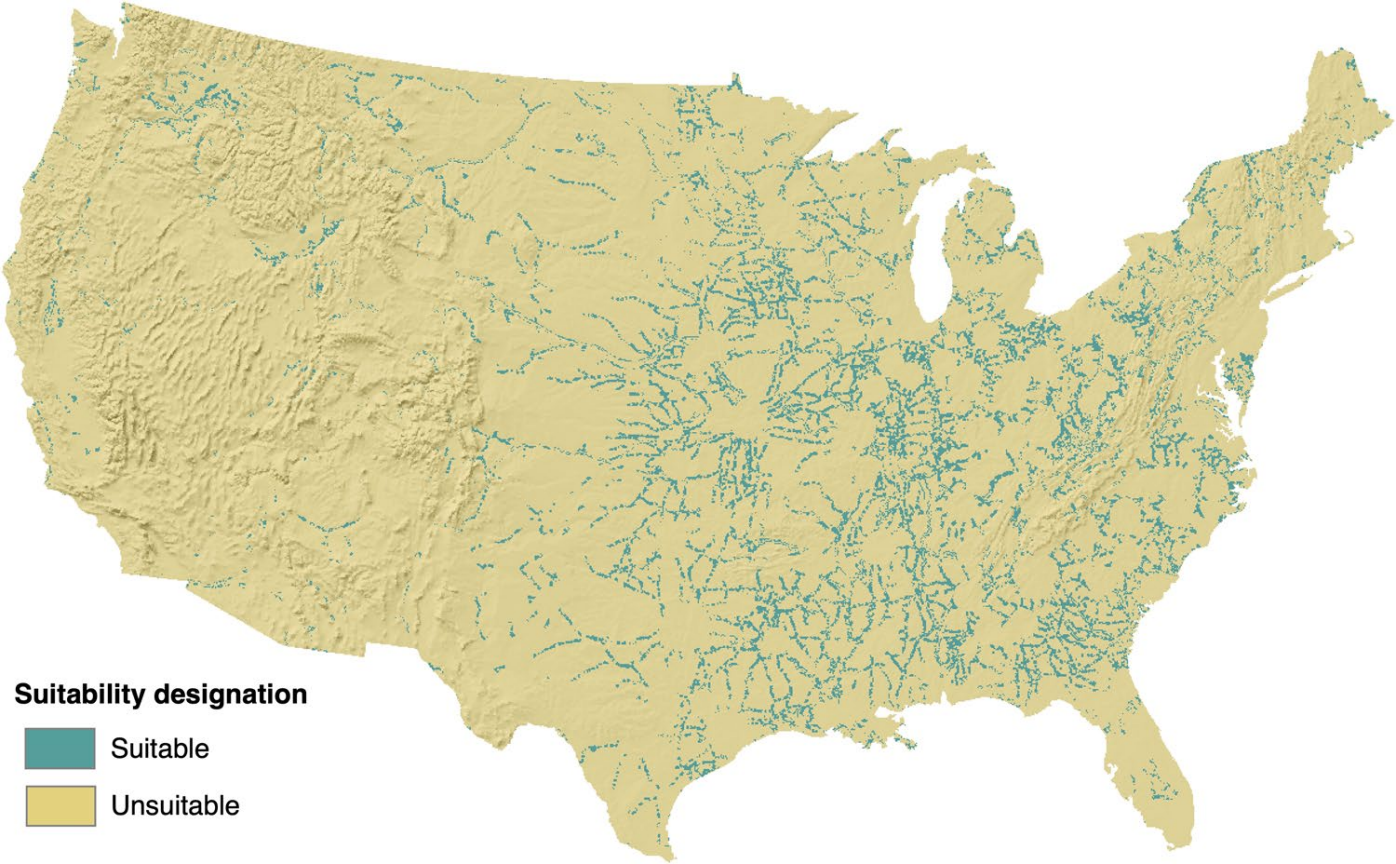
Technology-specific suitability for conventional biomass power plants that use recirculating cooling without carbon capture and storage. This suitability raster accounts for the common constraints that apply to thermoelectric suitability (Fig. 1) as well as additional constraints for distance to airports, rail nodes, navigable waters, densely populated areas, and available cooling water.

The GRIDCERF data package uses a portfolio of geospatial data over the conterminous U.S. (CONUS) to create land suitability layers tailored to the siting requirements of 56 renewable and non-renewable power plant technology configurations (Table 1). We use a common spatial resolution of 1 km^2^ (~250 acres) as a basis for the average footprint of a utility-scale power plant^10^. Renewable resources such as wind and solar have much lower power densities than non-renewables and therefore require more land to achieve the capacity of a utility-scale power plant^10^. We address the larger land footprint of wind and solar in multiples of 1 km^2^ grid cells. Each technology’s siting suitability layer represents a composite of geospatial information representing key siting constraints. Some technologies share certain siting constraints (e.g., avoidance of federally designated national wildlife refuges), and GRIDCERF facilitates creating a common suitability layer to which the user can add technology-specific constraint layers. For example, Fig. 1 shows the overall effect of the 20 common constraints identified for thermoelectric power plants on suitable land (see Table 2), and Fig. 2 shows the net result of adding technology-specific constraints for biomass power plant siting to this common layer. Individual layers may be static or dynamic to facilitate modeling of the future energy system under changing conditions. For example, energy system expansion models are typically based (in part) on population projections. Therefore, GRIDCERF contains dynamic population density layers built from high-resolution 21^st^ century projections of population^11^ to be used for technologies whose siting is influenced by proximal population densities.

**Table 1:** Power plant technology configurations represented in the GRIDCERF data package.

Technology	Sub-type	With or Without Carbon Capture Sequestration (CCS)	Cooling Type
Biomass	Conventional	CCS	D, OT, R
		No-CCS	D, OT, P, R
	Integrated Gasification Combined Cycle (IGCC)	CCS	D, OT, R
		No-CCS	D, OT, R
Coal	Conventional	CCS	D, OT, R
		No-CCS	D, OT, P, R
	IGCC	CCS	D, OT, R
		No-CCS	D, OT, R
Gas	Combined cycle (CC)	CCS	D, OT, R
		No-CCS	D, OT, P, R
	Combustion Turbine (CT)	No-CCS	D, OT, P, R
Nuclear	Gen 3	N/A	OT, P, R
Refined Liquids	CC	CCS	D, OT, R
		No-CCS	D, OT, R
	CT	No-CCS	D, OT, P, R
Solar	Concentrating Solar Power (CSP)	N/A	D
		N/A	R
	Photovoltaic (PV)	N/A	N/A
Wind	Onshore, 80 m	N/A	N/A
	Onshore, 110 m	N/A	N/A
	Onshore, 140 m	N/A	N/A

Cooling types are designated by Dry (D), Once-through (OT), Recirculating (R), Pond (P), and Not Applicable (N/A).

**Table 2:** Suitability layers common to all technologies.

**Common Suitability Layer Type and Source**		
Bureau of Land Management (BLM) Surface Management Agency Areas^50^	NPS Historic Trails^51^	U.S. Forest Service (USFS) Administrative Boundaries^52^
BLM National Landscape Conservation System (NLCS) - National Monuments^53^	NPS Scenic Trails^54^	USFS Wilderness Areas^55^
BLM NLCS - Outstanding Natural Areas^56^	U.S. Fish and Wildlife Service (USFWS) - Critical Habitat^57^	U.S. Geological Survey (USGS) National Wilderness Lands^58^
BLM NLCS - Wilderness^59^	USFWS - Special Designation^60^	USGS Protected Areas of the U.S - Class 1&2^36^
BLM NLCS - Wilderness Study Areas^61^	USFWS - Wild and Scenic River System^37^	U.S. State Protected Lands^62^
United States Environmental Protection Agency (EPA) Class 1 airsheds^63^	USFWS - National Realty Tracts^38^	Nature Conservancy lands^64^
National Park Service (NPS) Administrative Boundaries^65^	National Land Cover Dataset (NLCD) Wetlands^35^

While it is a standalone data package that can be utilized in geospatial analyses and energy planning, we designed GRIDCERF to provide the necessary inputs for models that simulate power plant siting for regional capacity expansion planning such as the Capacity Expansion Regional Feasibility (CERF)^12^ open-source software (see the Usage section of this paper for more detail). GRIDCERF is the only open-source data package providing centralized data to evaluate power plant siting suitability in a harmonized, rasterized format for use with energy system expansion planning models. Other existing tools, such as the Geospatial Energy Mapper^13^, do not provide data in a standardized format and are therefore incompatible with integrated, multi-model approaches to address the challenges of energy system expansion.

## Methods

### Definition of technologies

The GRIDCERF data package currently provides suitability data for most of the electricity generating technologies represented in the 50-state U.S. version of the Global Change Analysis Model (GCAM-USA)^14^ (Table 1). GCAM-USA technology types were derived from the U.S. Energy Information Administration (EIA) form EIA-860 that details generator-level data on both existing and planned generators, making these technologies widely applicable for U.S.-based analysis^15^. Most technologies also contain sub-types that can require additional consideration for suitability requirements. For example, we break out technologies into their cooling types (e.g., dry, recirculating) where applicable. The GRIDCERF data package does not currently support offshore wind, geothermal, distributed photovoltaic (PV), or hydropower technologies.

### Data planning, preparation, and processing

We developed a set of siting criteria for each technology type based on a literature review, current evaluations of actual power plant siting procedures as reported by the electric utility industry, and existing siting regulations^13,16,17^. These reviews revealed that many technologies share siting criteria. For example, physical features such as topography, critical habitat, and protected lands are common considerations when placing new power plants. In contrast, minimum mean annual flow requirements for thermoelectric cooling water are only relevant for thermoelectric power plants utilizing surface waters for cooling. GRIDCERF captures both common and specific types of siting constraints to a) broadly address the siting considerations of power plants and b) provide technology-specific data that can be extended or reduced to account for regionally relevant requirements.

The GRIDCERF data package currently contains 20 suitability layers considered to be common constraints for power plant siting (Table 2) and 24 categories of layers used to build technology-specific suitability layers (Table 3). Table 3 lists individual technology-specific layers in categories to be succinct because there are often multiple layers present for different variations suiting technology needs. GRIDCERF’s technology-specific layers also include dynamic layers, such as densely populated areas for the years 2020–2100 in 5-year timesteps based on the Shared Socioeconomic Pathways (SSPs)^11^. GRIDCERF also provides precompiled, common plus technology-specific layers for all 56 technology configurations utilizing the population projections for 2020 from SSP 2 (a middle of the road scenario representing socioeconomic and technological trends that are not notably different from historical patterns^18^). Some GRIDCERF technology-specific layers, such as the United States Environmental Protection Agency (EPA) National Ambient Air Quality Standards non-attainment zones for a variety of pollutants, are not included in the default configuration for the precompiled layers but are available to address specific research objectives. Note that most of the sources listed in Tables 2, 3 do not natively provide datasets archived with a Digital Object Identifier (DOI), but the GRIDCERF data package does to ensure permanence for the rasters we have derived from these sources. All source licenses and/or disclaimers and retrieval metadata are housed in GRIDCERF’s data archive.

**Table 3:** Suitability layers available for custom technology-specific compilation.

**Technology-specific Suitability Layer Type and Source**		
Bureau of Indian Affairs (BIA) Land Area Representations Dataset^66^	Coal Supply^41–43^	Earthquake Potential^39^
Slope 5% or less suitable^21^	United States Environmental Protection Agency (EPA) CO Non-attainment Areas^67^	Densely populated areas^11^
Slope 10% or less suitable^21^	EPA NOx Non-attainment Areas^67^	Densely population areas buffered by 25 miles^11^
Slope 12% or less suitable^21^	EPA Ozone Non-attainment Areas^67^	Densely population areas – nuclear^11^
Slope 20% or less suitable^21^	EPA Lead Non-attainment Areas^67^	National Hydrography Dataset (version 2; NHDv2)^34^
Airports (10-mile buffer)^68^	EPA PM10 Non-attainment Areas^67^	National Renewable Energy Laboratory (NREL) concentrating solar direct normal potential^27^
Airports (3-mile buffer)^68^	EPA PM2.5 Non-attainment Areas^67^	NREL photovoltaic potential^27^
Proximity to Railroad and Navigable Waters (<5 km)^41,42^	EPA SOx Non-attainment Areas^67^	NREL Wind Integration National Dataset (WIND) toolkit wind development potential^23^

The spatial data sources in Tables 2, 3 include both raster and vector (e.g., line, polygon, and point) data of varying resolutions and coordinate reference systems. Descriptions, sources, processing steps and/or Python code for converting the raw data we harvested to the rasters produced in GRIDCERF are provided in Jupyter notebooks as hosted in our GitHub repository for this publication (see Code Availability section). The steps for the processing of each type of our data are listed as follows. To ensure consistency and interoperability, we first transformed all spatial data into the USA Contiguous Albers Equal Area Conic^19^ (ESRI:102003) projected coordinate system. This meter-based system uses the North American Datum of 1983 (NAD 83). We employed bilinear interpolation to perform any necessary raster resampling to transform continuous data from its native coordinate system to our target system. We determined the new cell value using the weighted distance average of the four nearest grid cells. Raster resampling required for discrete data utilized a nearest neighbor assignment^20^.

For datasets requiring downscaling or upscaling for discrete data, we utilized a nearest neighbor assignment^20^ when downscaling and the majority grid cell value when upscaling. For continuous data requiring aggregation, we used either a sum or the mean of the encompassed grid cells, depending on the type of data being processed (that is, sum for data where each grid cell value contributes to the total of the coarser grid cell and mean where the individual contributions are relative to the behavior of the coarser grid cell). Vector data sources were rasterized using an allocation method that designated a grid cell with the value attribute of the geometry if the feature topologically touched the 1 km^2^ grid cell.

Next, we reclassified each raster to binary values where 0 indicates a suitable grid cell and 1 indicates an unsuitable grid cell. This enables users to investigate how a particular criterion affects the technical feasibility of siting across the model’s geographic domain. GRIDCERF rasters can be summed as a stack (or multidimensional array) where suitability remains equal to 0 and unsuitable grid cells sum to their number of unsuitable constraints. Users can then quickly assess which constraints may be siting barriers in a region of interest. Grid cells labeled unsuitable by a constraint subject to mitigating action considerations (e.g., wetlands) in the region of interest can be removed or replaced by a different, locally relevant constraint. The code provided with the GRIDCERF data package can automatically generate compiled rasters based on user-defined layer needs. The following describes the processing steps for layers having specific specialized workflows. All other layers had non-specialized processing workflows and followed the process for conversion to the GRIDCERF required format described above.

We built our slope suitability layers for each technology using Shuttle Radar Topography Mission 90-meter elevation data^21^ via the Google Earth Engine. We converted the native output of slope in degrees from the Earth Engine “Terrain.slope” function into percent slope, then reprojected and resampled the output into GRIDCERF’s native resolution. We then reclassified the percent slope data into GRIDCERF’s binary format for varying levels of slope suitability as required by each technology. GRIDCERF technology-specific slope requirements are described in their respective sections.

Several technologies require a set distance from densely populated areas. For our population layers, we processed projections of the CONUS population through the end of the century under the SSPs by mosaicking the state-specific rasters from Zoraghein and O’Neill^11^ at a 1 km^2^ resolution and applying a maximum value filter if state boundaries overlapped. These data were only available for SSP 2, 3, and 5 in 10-year time-steps. We conducted a linear interpolation to accommodate the smaller timestep of models such as GCAM-USA and provide the SSP data at a 5-year timestep. We then calculated three different suitability layers (unbuffered, buffered, and nuclear specific) for each available SSP and year. Unbuffered densely populated area rasters represent areas of at least 50,000 inhabitants within an area of 25 square miles as unsuitable. Buffered population density rasters were built to represent industry assumptions for certain technologies (e.g., coal) with a recommended distance of 25 miles to a densely populated area. Finally, we built a nuclear-specific population density raster built to account for Nuclear Regulatory Commission regulations that require nuclear power plants to establish exclusion and low population zones around their perimeter based on exposure pathways in case of a radiation release. We constructed this layer to represent common guidelines outlined by the Electric Power Research Institute for meeting those regulations, which advise that a potential site is unsuitable if the population density is greater than 500 people per square mile for any radius out to 20 miles^22^.

For wind resource suitability, we harvested criteria and source data from the National Renewable Energy Laboratory (NREL) Wind Integration National Dataset (WIND) toolkit^23–25^. Onshore wind resources were derived using potential wind capacity considering 80-, 110-, and 140-meter hub heights for current technology designations. We based our suitability screening on the potential to meet the requirements of a utility scale power plant. We designated areas as suitable for onshore wind by determining which 1 km^2^ areas could achieve a 35% capacity factor or greater using wind potential data after land area exclusions were applied. This capacity factor chosen was intended to align with GCAM-USA assumptions for CERF modeling^26^. We aggregated these data from 200 m^2^ resolution to our 1 km^2^ standard. Note that wind development can be pursued in areas that achieve a lower capacity factor and that we have provided the code (see Code Availability) necessary to produce additional layers using a less conservative estimate which will open additional suitable areas for development.

For solar resource suitability, we utilized the NREL National Solar Radiation Database^27^ (NSRDB). We determined utility-scale solar PV resource availability using the State University of New York/Albany (SUNY) satellite radiation model’s annual average latitude equals tilt irradiance. For Concentrating Solar Power (CSP), we utilized the SUNY annual average indirect normal irradiance data^27^. We evaluated utility-scale PV resource potential suitability assuming an average power plant size of 75 MW with a capacity factor of 23%^26,28^ which we validated using existing solar PV installations (see Technical Validation section). We determined the irradiance level (power per unit of area) by backing out the irradiance required to achieve the assumed capacity factor of the utility-scale PV resource while also accounting for the ratio between the array’s DC rated size and the inverter’s AC rated size, equal to 1.3^29^. We categorized grid cells having average irradiance values greater than or equal to 4.246 kWh m^−2^ d^−1^ as capable of supporting a centralized PV power plant. Grid cells with annual average direct normal irradiance values greater than or equal to 4.615 kWh m^−2^ d^−1^ based on the SUNY model outputs are considered capable of supporting CSP power plants rated at 100 MW with a capacity factor of 25%. We further refined the suitability criteria for solar and wind by including GRIDCERF’s common suitability layers for the technical potential data for PV and CSP. We defined suitable slopes as 10% or less for PV and 5% or less for CSP^13,30^.

Thermoelectric technologies have minimum mean annual flow requirements for cooling water specified in the Clean Water Act (CWA) 316(b)^31^. CWA 316(b) specifies that all power plants that withdraw over 2 million gallons per day (MGD) from a freshwater river or stream must withdraw less than five percent of the source water’s mean annual flow. Additionally, plants requiring over 10 MGD that cannot reduce their intake flow to a closed-cycle recirculating system are no longer permitted^32^. To determine suitable geospatial locations for technologies that fall between the two levels, we calculated the required minimum mean annual flow based off water withdrawal intensity (m^3^/MWh) estimates from Macknick *et al*.^33^, the assumed investment-level capacity factor for each technology (%), and the assumed power plant unit size (MW) for each technology. We used the results from our 56 thermoelectric technology types (inclusive of technology, sub-technology, and cooling type information) to create 13 total bins of mean annual flow. The binned values range from >2 MGD to >145 MGD. We used each minimum stream flow bin to select flowlines (line geometry features containing flow attributes) from the NHDPlus Version 2 data meeting the criteria of the bin^34^. We buffered the flowlines by 20 km to represent the reasonable distance necessary to utilize the water source for cooling and then rasterized them to GRIDCERF’s resolution of 1 km^2^. We define cooling water suitability as grid cells that have sufficient flow within the buffered area from the waterway.

We generated our wetlands suitability data using the National Land Cover Database (NLCD)^35^ 2019 30 m raster data. The NLCD raster was resampled to upscale the 30 m resolution to GRIDCERF’s 1 km resolution using the majority grid cell value of the finer resolution to determine the value of the coarser resolution. The data was then reclassified to set NLCD Class 90 (woody wetlands) and Class 95 (emergent herbaceous wetlands) as unsuitable. The reclassified raster was then converted to a polygon shapefile where each polygon represented wetland areas. We then used the general rasterization process described above for consistency to produce the suitability raster.

Several other layers required specific filtering and/or spatial buffering steps to process their data into the GRIDCERF format. For the Protected Areas Database of the United States (PAD-US)^36^ we used only the GAP status classes 1 and 2 to account for managed biodiversity lands. United States Fish and Wildlife Service (USFWS) wild and scenic rivers^37^ and the USFWS national realty tracts^38^ were buffered by 1 km to maintain connectivity. We developed the earthquake potential suitability layers from the National Seismic Hazard Model^39^ data by specifying a peak ground acceleration (PGA) of 3.5 g or greater at probability levels of two percent in 50 years as unsuitable for nuclear siting^40^. Our proximity to railroad and navigable waters suitability layer combined U.S. Department of Transportation (USDOT) Bureau of Transportation Statistics (BTS) North American rail network nodes^41^ and USDOT BTS navigable waterway network lines^42^ after a buffer of 5 km was applied as a proxy for an access zone to these resources. Similarly, the coal supply layer combined the rail nodes and navigable waterway buffered data with the Energy Information Association (EIA) U.S. Coal Mining Locations^43^ data buffered by 20 km to proxy as an access zone to mining locations.

## Data Records

All rasters in the GRIDCERF package are available in GeoTIFF format at a 1 km^2^ resolution. The full data package is published on Zenodo and is compressed into a zipped file for download^17^. We also included documents in the reference directory of the archive that detail the individual layers used to compile the final 56 technology suitability layers as well as an additional literature review document that was used to evaluate the types of technology-specific considerations necessary to include in our analysis. GRIDCERF is available under the Creative Commons Attribution 4.0 International license.

## Technical Validation

We validated the suitability data presented in this paper using publicly available power plant data and locations distributed by the EIA^44^. The power plant data period covered through April 2020 and was last updated on July 7, 2020. We also utilized form EIA-923 to append the operational year to each power plant^15^. We perform the validation in the following three categories, non-renewable technologies, onshore wind, and solar PV, due to their significant differences in suitability criteria.

### Non-renewable technologies

For non-renewable technologies, we selected operational electricity generating plants from the EIA data that: (1) have a combined nameplate capacity of 80 MW or greater to represent utility-scale plants, (2) are located in the CONUS, and (3) became operational during or after the year 2000 to represent modern planning considerations for power plant siting. The technology mix of the 497 qualifying power plants consisted of natural gas (94.8%), coal (3.6%), petroleum (1%), and biomass (0.6%).

We then determined how many of the qualifying power plants were sited on suitable versus unsuitable grid cells according to GRIDCERF. Non-renewable sites were validated against suitability built from a combination of GRIDCERF’s common exclusion layer set (see Table 2), and a slope suitability layer where slopes steeper than 12% are deemed unsuitable^45,46^. We found 107 power plants, or 22%, that violated this combination of layers. The technology-specific breakdown of invalid plants is as follows: 101 natural gas, four petroleum, two coal, and no biomass plants. We determined the GRIDCERF National Park Service (NPS) historic trails layer contributed to 36% of the violations, and that the violations were spatially heterogenous, spanning 35 states. U.S. state protected lands and U. S. Fish and Wildlife Service (USFWS) critical habitat contributed to 30% and 21% of the violations, respectively. Other layers also minorly contributed to overall violations (Table 4).

**Table 4:** Layers causing violations for the non-renewable power plant validation.

Layer	Number Invalid	Percent Contribution to Total Violations
NPS Historic Trails	38	36
U.S. State Protected Lands	32	30
USFWS Critical Habitat	23	21
BLM Surface Management Agency Areas	14	13
USFWS National Realty Tracts	8	7
NPS Scenic Trails	7	7
NLCD Wetlands	4	4
Slope 12% or less suitable	3	3
Nature Conservancy lands	3	3
USGS Protected Areas of the U.S - Class 1&2	3	3
USFWS - Wild and Scenic River System	1	1
NPS Administrative Boundaries	1	1

Includes the number of power plants in the validation set that were in unsuitable grid cells as well as the percentage of invalid power plants per layer to the total number of violations. This table only includes layers having greater than zero violations.

**Fig. 3:**
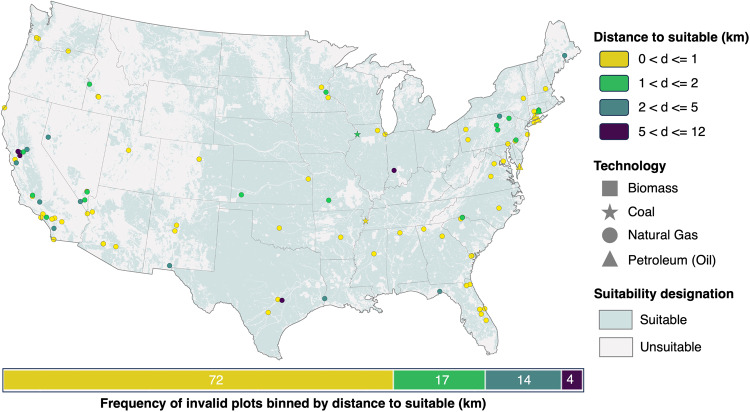
The spatial distribution of invalid non-renewable power plants by technology and distance to their nearest suitable grid cell.

Further investigation into the non-renewables siting violations shows that 72 out of 104 violations (69%) were within 1 km of a suitable grid cell and 89 (86%) were within 2 km (Fig. 3). Overall, the distance of invalid power plants to suitable grid cells had a Root Mean Square Error (RMSE) of 2.31 km, showing that power plants within our unsuitable areas were generally very close to suitable areas. Given that the goal of GRIDCERF is to identify feasible locations for new power plants at regional scale (as opposed to determining optimal locations for individual power plants), we believe these results validate our approach.

### Wind

For the wind siting validation, we selected operational wind farms from the EIA data that: (1) have a combined nameplate capacity of 3 MW or greater to represent utility-scale expansion for siting in a 1 km^2^ footprint, (2) are in the CONUS, (3) have a capacity factor of 35% or greater, and (4) became operational during or after the year 2000 to represent modern planning considerations for power plant construction. We validated wind sites against suitability criteria built from a combination of wind development potential for turbines with a hub height of 80 m and an assumed capacity factor of 35%, GRIDCERF’s common exclusion layer set (see Table 2), and a slope suitability layer of 0 to 20%, which represents a midrange of topography accessible for installation and maintenance^47^.

We evaluated a total of 378 wind installations of which 325 (86%) were co-located with suitable grid cells and 53 (14%) were co-located with unsuitable grid cells. We found that the primary contributor to wind suitability violations was the USFWS national realty tracts layer, which contributed to 36% of the violations. These violations were spread over nine states. Our layers for BLM surface management agency areas, USFWS critical habitat designated area, NPS historic trails, and U.S. state protected lands contributed to 26%, 17%, 15%, and 9% of violations, respectively. Other layers also minorly contributed to overall violations (Table 5).

**Table 5:** Layers causing violations for the wind power plant validation.

Layer	Number Invalid	Percent Contribution to Total Violations
USFWS National Realty Tracts	19	36
BLM Surface Management Agency Areas	14	26
USFWS Critical Habitat	9	17
NPS Historic Trails	8	15
U.S. State Protected Lands	5	9
Slope 20% or less suitable	3	6
NREL WIND toolkit: hub height 80 meters	2	4
USFS Administrative Boundaries	2	4
Densely populated areas	2	4
NPS Scenic Trails	1	2

Includes the number of power plants in the validation set that were in unsuitable grid cells as well as the percentage of invalid power plants per layer to the total number of violations. This table only includes layers having greater than zero violations.

Though critical habitat and other land and trail-designated land play a role in preferentially siting wind turbines, incidental take permits and Habitat Conservation Plans (HCP) can be filed to override this requirement^48^ (structurally similar to wetlands mitigation banking). We chose to include these restrictions in GRIDCERF to demonstrate how land could be developed based on regionally applicable restrictions. Similarly, we chose a slope suitability threshold (< = 20%) that provides a reasonable balance between what is highly preferential (< = 7%) and unreasonable (>40%) for accessibility^49^. This decision caused our slope suitability layer to contribute to 6% of violations. Relaxing this requirement would allow for wind to be sited in most of these areas.

**Fig. 4:**
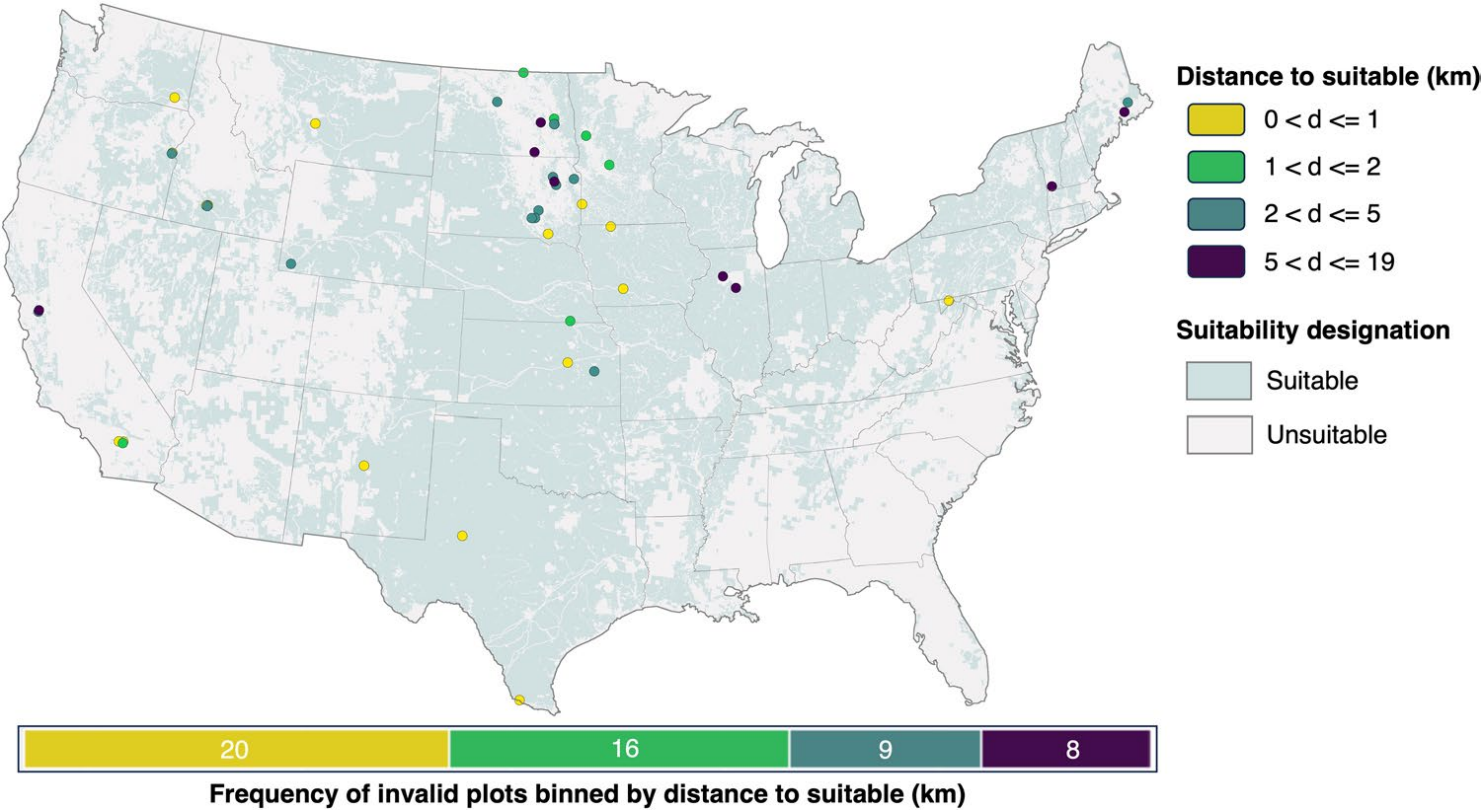
The spatial distribution of invalid wind installations and the distance to their nearest suitable grid cell.

We found that 20 (38%) of the 53 violations were within 1 km of a suitable grid cell and all but 8 (85%) were within 5 km of a suitable grid cell (Fig. 4). This resulted in a total RMSE of 4.42 km for all violations. The 8 violations greater than 5 km away from a suitable grid cell were generally in areas surrounded by USFWS managed land and/or critical habitat. Given the larger overall footprint needed for utility-scale wind as compared to utility-scale non-renewables, we believe these wind validation results support the use of GRIDCERF to understand potential regional scale wind expansion.

### Solar

For solar PV technologies, we selected operational plants from the EIA^44^ data that: (1) have a combined nameplate capacity of 50 MW or greater to represent utility-scale plants^14^, (2) are located in the CONUS, (3) became operational during or after the year 2000 to represent modern planning considerations for power plant siting, and (4) have a capacity factor of at least 23%. The EIA data contained solar PV sites, installed capacity (MW), and estimated annual generation information. We calculated annual mean generation for complete years over 2015–2021 for operational sites from EIA-923 data as representative generation of long-term operational behavior. The AC capacity factor for each installed PV plant was estimated from the installed capacity of the plant and annual mean generation. Solar PV sites were validated against suitability built from a combination of the previously discussed potential capacity layer, GRIDCERF’s common exclusion layer set (see Table 2), and a slope suitability layer where slopes steeper than 10% were deemed unsuitable as previously mentioned.

**Fig. 5:**
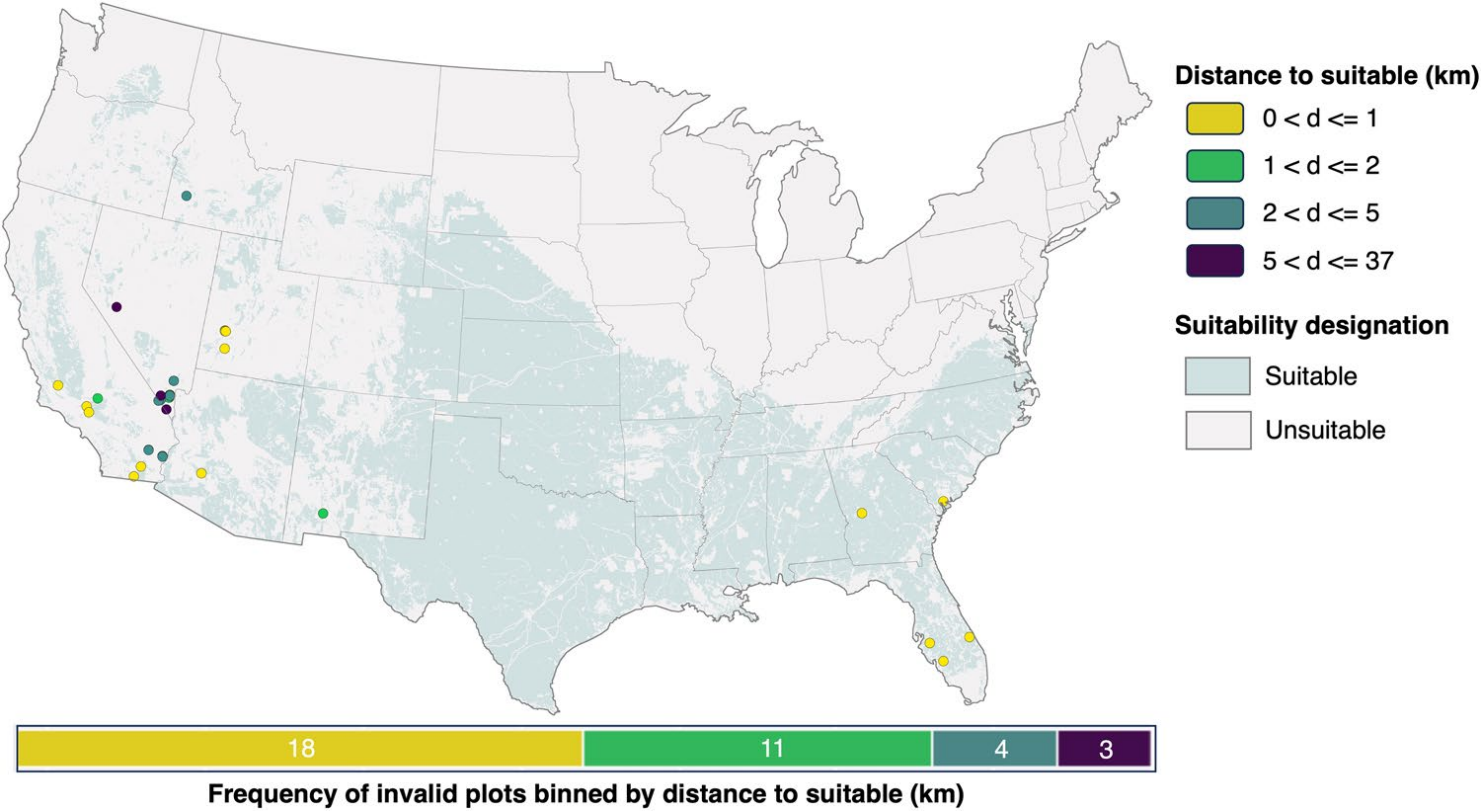
The spatial distribution of invalid solar PV installations and the distance to their nearest suitable grid cell.

A total of 154 EIA solar PV sites met our qualifying criteria for the solar PV siting validation. Of these, 118 (77%) were co-located with suitable grid cells and 36 (23%) were co-located with unsuitable grid cells. The layer with the largest contribution to violations (interacted with 58% of invalid sites) was the BLM surface management agency areas, with 21 invalid sites. Seven (19% of invalid) of the violations were from interactions with the NPS historic trails and five (14% of invalid) were from interactions with the U.S. state protected lands. Overall, 18 (50%) of the invalid solar PV sites were within 1 km of a suitable grid cell and an additional 11 (31%) were between 1 and 2 km of a suitable grid cell. The total RMSE for all violations was 7.32 km from a suitable grid cell (Fig. 5). As with the other validation analyses, we observed violations for constraints that are often mitigated or flexed on a site-specific basis (Table 6). We believe these solar validation results support the use of GRIDCERF to understand potential regional scale solar PV expansion.

**Table 6:** Layers causing violations for the solar PV power plants validation.

Layer	Number Invalid	Percent Contribution to Total Violations
BLM Surface Management Agency Areas	21	58
NPS Historic Trails	7	19
U.S. State Protected Lands	5	14
USFWS Critical Habitat	3	8
Slope 10% or less suitable	2	6
BLM NLCS - National Monuments	2	6
USGS Protected Areas of the U.S - Class 1&2	1	3
NPS Scenic Trails	1	3
USFWS National Realty Tracts	1	3
NLCD Wetlands	1	3

Includes the number of power plants in the validation set that were in unsuitable grid cells as well as the percentage of invalid power plants per layer to the total number of violations. This table only includes layers having greater than zero violations.

## Usage Notes

We initially developed the GRIDCERF data package for direct use with the Capacity Expansion Regional Feasibility (CERF) open-source modeling software^12^ (available as a Python package) but found that the data could be broadly applicable to modeling power plant expansion. The CERF software was constructed to be coupled to a multisectoral global change model (GCAM-USA) that produces energy system expansion scenarios at the U.S. state scale given a set of policy, climate, technology change, resource availability, and socioeconomic drivers. Its goal is to determine feasible individual power plant locations at a 1 km^2^ grid cell resolution for GCAM-USA’s energy system expansion plans. The CERF software uses an economic algorithm to select individual sites deemed suitable from the tailorable GRIDCERF data package suitability layers. In short, the algorithm distributes power plants across the suitability landscape by causing power plants to compete for suitable siting locations based on their grid interconnection costs and on the locational value of their power generation. The data presented in this paper make it possible to conduct this type of analysis and allow customization driven by science questions.

## Data Availability

The code used to build the data described in the GRIDCERF package can be accessed on GitHub here: https://github.com/IMMM-SFA/vernon-etal_2023_scidata. They are in a Jupyter Notebook format to allow for interactive visualization and description throughout. The CERF modeling software is also available at https://github.com/IMMM-SFA/cerf and is detailed in Vernon *et al*.^12^.
